# Supplementary material for: Microphthalmia-Associated Transcription Factor: A Differentiation Marker in Uveal Melanoma
Source: Int J Mol Sci. 2023 May 16;24(10):8861. doi: 10.3390/ijms24108861 (PMC10218684; doi:10.3390/ijms24108861)
Supplement: Supplementary file 1 [file ijms-24-08861-s001.zip › Supplementary Table S1.pdf]

Supplementary Table S1. Correlations between expression of MITF and cytokines in 64 UM patients from the Leiden cohort (microarray). Spearman's correlation. MITF expression (Mean, ± SD) = 10.46 (±0.5)

	Mean (±SD)	Correlation Coeff.	Sig (2-tailed)
CCL2	7.37 (±0.7)	-0.119	0.35
CCL5 pr1	7.53 (*±1.2)*	-0.371	0.003
CCL5 pr2	8.52 (*±1.5)*	-0.328	0.008
CXCL10	7.36 (*±1.0)*	-0.267	0.033
IL10	9.56 (*±0.6)*	-0.105	0.41
