# Supplementary material for: Microphthalmia-Associated Transcription Factor: A Differentiation Marker in Uveal Melanoma
Source: Int J Mol Sci. 2023 May 16;24(10):8861. doi: 10.3390/ijms24108861 (PMC10218684; doi:10.3390/ijms24108861)
Supplement: Supplementary file 1 [file ijms-24-08861-s001.zip › Supplementary Table S2.pdf]

Supplementary Table S2: top 5 upregulated and top 5 downregulated genes in UM with low MITF vs UM with high MITF in the Leiden cohort.

	Symbol	Entrez	logFC	AveExpr	t	P.Value	adj.P.Val	B	Location	Description
Upregulated in MITF loss	CXCL16	58191	0.741486	8.413755	4.460757	3.36E-05	0.009452	2.205388	17p13.2	C-X-C motif chemokine ligand 16
	RMDN1	51115	0.649212	8.273728	4.421381	3.86E-05	0.010057	2.079305	8q21.3	Regulator of microtubule dynamics 1
	HAVCR2	84868	0.792435	8.250183	4.331454	5.3E-05	0.011966	1.793475	5q33.3	Hepatitis A virus cellular receptor 2 (TIM3)
	CNIH4	29097	0.642492	10.1966	4.321953	5.48E-05	0.011966	1.763453	1q42.11	Cornichon family AMPA receptor auxiliary protein 4
	HCP5	10866	1.551341	9.148227	4.133257	0.000106	0.014577	1.174445	6p21.33	HLA complex P5
Downregulated in MITF loss	IRS2	8660	-0.70351	9.537433	-7.10912	1.17E-09	1.89E-05	11.48943	13q34	Insulin receptor substrate 2
	AHCYL2	23382	-1.18313	9.329294	-5.44432	8.71E-07	0.001646	5.507751	7q32.1	Adenosylhomocysteinase like 2
	PPP1R3C	5507	-1.20628	8.913484	-4.65239	1.69E-05	0.006196	2.826651	10q23.32	Protein phosphatase 1 regulatory subunit 3c
	SNHG7	84973	-0.92191	11.95	-4.28843	6.16E-05	0.012044	1.657804	9q34.3	small nucleolar RNA host gene 7
	CDV3	55573	-0.60191	8.652244	-4.22338	7.73E-05	0.012546	1.454012	3q22.1	CDV3 homolog
