# Supplementary material for: Microphthalmia-Associated Transcription Factor: A Differentiation Marker in Uveal Melanoma
Source: Int J Mol Sci. 2023 May 16;24(10):8861. doi: 10.3390/ijms24108861 (PMC10218684; doi:10.3390/ijms24108861)
Supplement: Supplementary file 1 [file ijms-24-08861-s001.zip › Supplementary Table S3.pdf]

**Supplementary Table S3:** list of Illumina probes used for each gene examined (HT-12 v4 chip)..

| GENE | PROBE |
|---|---|
| MITF | ILMN_2304186 |
| MC1R | ILMN_1653319 |
| MLANA | ILMN_1710217 |
| PMEL | ILMN_1665994 |
| TYR | ILMN_1788774 |
| TYRP1 | ILMN_2054652 |
| Rab27a pr1 | ILMN_2329773 |
| Rab27a pr3 | ILMN_1665859 |
| OA1 | ILMN_1756261 |
| SOX10 | ILMN_1653750 |
| SNAI2 pr1 | ILMN_1655740 |
| SNAI2 pr2 | ILMN_2082585 |
| ZEB2 | ILMN_1688698 |
| TWIST1 | ILMN_1672908 |
| STAT3 pr1 | ILMN_1663618 |
| STAT3 pr2 | ILMN_2401978 |
| CD3D | ILMN_2261416 |
| CD3E | ILMN_1739794 |
| CD4 | ILMN_1727284 |
| CD8A pr1 | ILMN_1768482 |
| CD8A pr3 | ILMN_2353732 |
| CD68 | ILMN_1714861 |
| CD163 | ILMN_1733270 |
| HLA-A pr1 | ILMN_1671054 |
| HLA-A pr2 | ILMN_2203950 |
| HLA-A pr4 | ILMN_2186806 |
| HLA-B | ILMN_1778401 |
| HLA-DR pr1 | ILMN_1689655 |
| HLA-DR pr2 | ILMN_2157441 |
