# Supplementary figures and images for: Microphthalmia-Associated Transcription Factor: A Differentiation Marker in Uveal Melanoma
Source: Int J Mol Sci. 2023 May 16;24(10):8861. doi: 10.3390/ijms24108861 (PMC10218684; doi:10.3390/ijms24108861)

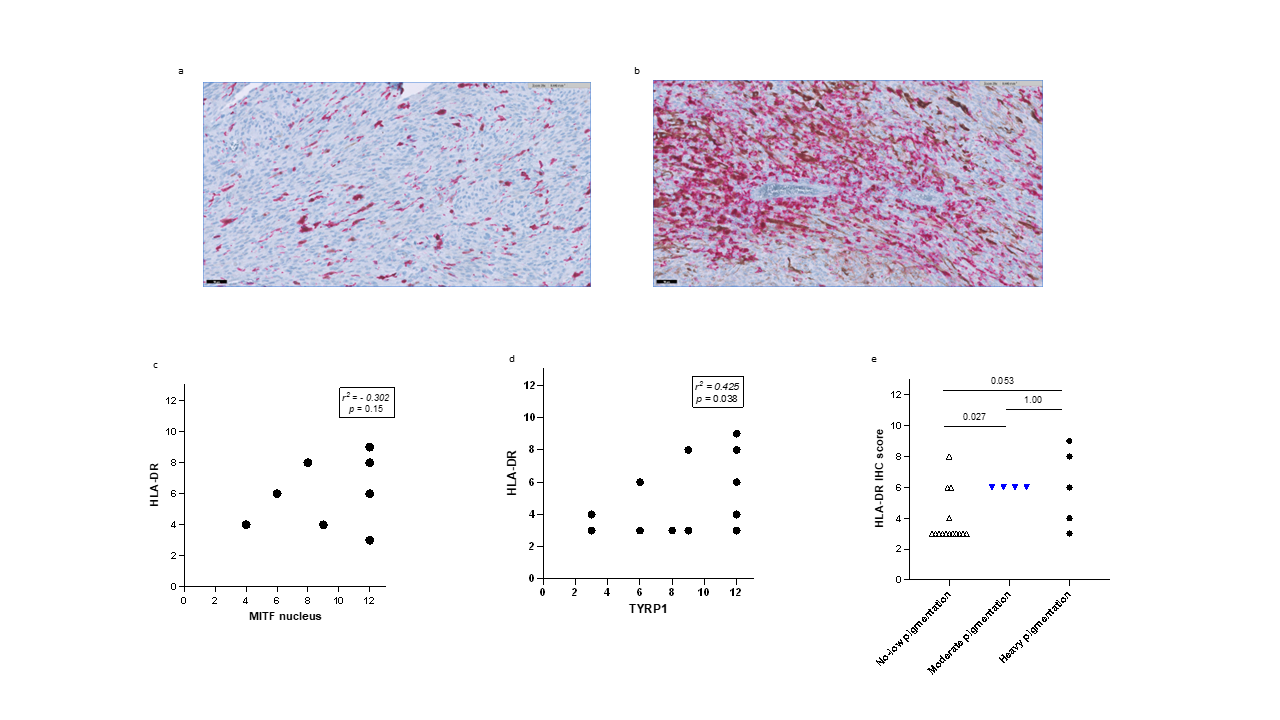

Supplement: Supplementary file 1 [file ijms-24-08861-s001.zip › Maria Chiara Gelmi - Figure S1 MITF.tif]

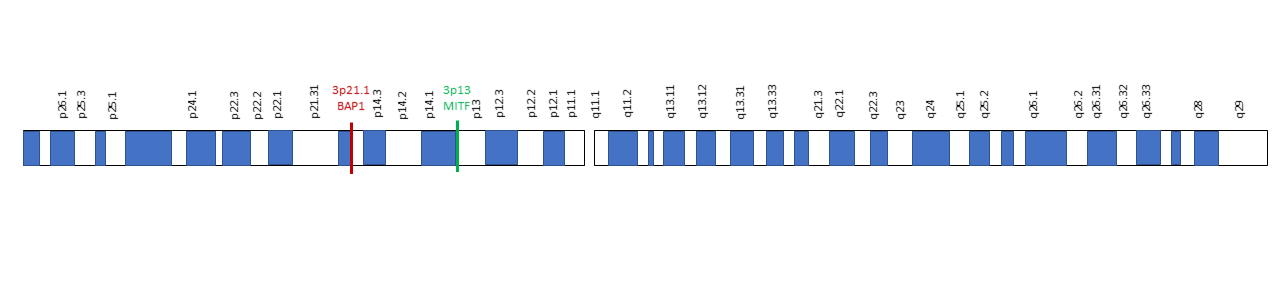

Supplement: Supplementary file 1 [file ijms-24-08861-s001.zip › Maria Chiara Gelmi - Figure S2 MITF.tif]
